# Documentation of ethically relevant information in out-of-hospital resuscitation is rare: a Danish nationwide observational study of 16,495 out-of-hospital cardiac arrests

**DOI:** 10.1186/s12910-021-00654-y

**Published:** 2021-06-30

**Authors:** Louise Milling, Lars Grassmé Binderup, Caroline Schaffalitzky de Muckadell, Erika Frischknecht Christensen, Annmarie Lassen, Helle Collatz Christensen, Dorthe Susanne Nielsen, Søren Mikkelsen, The Danish Cardiac Arrest Registry Group, René Arne Bergmann, Stig Nikolaj Fasmer Blomberg, Lars Borup, Mathias Geldermann Holgersen, Theo Walther Jensen, Gunhild Kjærgaard-Andersen, Julie Linding Bogh Kjerulff, Heinrich Dedenroth Larsen, Kenneth Lübcke, Kristian Bundgaard Ringgren

**Affiliations:** 1 Prehospital Research Unit, Department of Anaesthesiology and Intensive Care, Odense University Hospital, Kildemosevej 15, 5000 Odense C, Denmark; 2 Department of Regional Health Research, University of Southern Denmark, Odense, Denmark; 3 Philosophy, Department for the Study of Culture, University of Southern Denmark, Odense, Denmark; 4 Centre for Prehospital and Emergency Research, Aalborg University Hospital, Aalborg University, Aalborg, Denmark; 5 Emergency Medicine Research Unit, Odense University Hospital, Odense, Denmark; 6 The Danish Clinical Quality Program, National Clinical Registries, Copenhagen, Denmark; 7 Department of Infectious Diseases, Sub-department of Immigrant Medicine, Odense University Hospital, Odense, Denmark; 8 Department of Geriatric Medicine, Odense University Hospital, Odense, Denmark

**Keywords:** Bioethics, Cardiac arrest, Resuscitation, Emergency medical services, Decision-making

## Abstract

**Background:**

Decision-making in out-of-hospital cardiac arrest should ideally include clinical and ethical factors. Little is known about the extent of ethical considerations and their influence on prehospital resuscitation. We aimed to determine the transparency in medical records regarding decision-making in prehospital resuscitation with a specific focus on ethically relevant information and consideration in resuscitation providers’ documentation.

**Methods:**

This was a Danish nationwide retrospective observational study of out-of-hospital cardiac arrests from 2016 through 2018. After an initial screening using broadly defined inclusion criteria, two experienced philosophers performed a qualitative content analysis of the included medical records according to a preliminary codebook. We identified ethically relevant content in free-text fields and categorised the information according to Beauchamp and Childress’ four basic bioethical principles: autonomy, non-maleficence, beneficence, and justice.

**Results:**

Of 16,495 medical records, we identified 759 (4.6%) with potentially relevant information; 710 records (4.3%) contained ethically relevant information, whereas 49 did not. In general, the documentation was vague and unclear. We identified four kinds of ethically relevant information: patients’ wishes and perspectives on life; relatives’ wishes and perspectives on patients’ life; healthcare professionals’ opinions and perspectives on resuscitation; and do-not-resuscitate orders. We identified some “best practice” examples that included all perspectives of decision-making.

**Conclusions:**

There is sparse and unclear evidence on ethically relevant information in the medical records documenting resuscitation after out-of-hospital cardiac arrests. However, the “best practice” examples show that providing sufficient documentation of decision-making is, in fact, feasible. To ensure transparency surrounding prehospital decisions in cardiac arrests, we believe that it is necessary to ensure more systematic documentation of decision-making in prehospital resuscitation.

**Supplementary Information:**

The online version contains supplementary material available at 10.1186/s12910-021-00654-y.

## Background

In most parts of North America, guidelines exist that allow paramedics to decide on the termination of resuscitation without consulting a physician in the treatment of out-of-hospital cardiac arrest (OHCA) patients [1, 2]. In most European countries, however, healthcare professionals must make this decision relying on medical judgment [3, 4]. The exigent nature of the decisions and scarcity of information complicate this decision process. In-hospital healthcare professionals often deliberate with peers and gain insights from medical specialists or medical records in these situations, whereas prehospital healthcare professionals usually are a small team present at the scene. In countries with prehospital physicians, the physician often has decision-making authority and is solely responsible for these decisions. The physician may include the ambulance crew in the decision, but in a study from Duchateau et al. concerning the withholding of cardiopulmonary resuscitation (CPR), the authors conclude that the physicians only do so in 48% of cases [5]. In countries without prehospital physicians, or in situations where the prehospital physician may not be readily available, paramedics, emergency medical technicians, and prehospital nurses face similar decision-making [6, 7]. This is the case in Sweden where prehospital nurses face challenging decision-making [8]. Decision-making in prehospital resuscitation should ideally include both clinical and ethical considerations [9]. There is not much knowledge of the extent and quality of ethical considerations and their influence on resuscitation [10, 11]. The European Resuscitation Council guidelines include the sub-category “Ethics of resuscitation and end of life decisions”. These guidelines emphasise that “clinicians should explore and understand the value that a patient places on specific outcomes” and underline the importance of documenting reasons for resuscitation decisions [12]. In this study, we explored the extent, characteristics, and transparency of documented ethically relevant information in OHCA based on a review of prehospital medical records. We assessed the amount of ethical information in prehospital medical records and characterised the content according to bioethical principles.

## Methods

### Design

This Danish nationwide study investigated the ethically relevant information documented in prehospital emergency medical records in three steps. We included the medical records from all OHCA patients from 2016 to 2018 and used a broad definition of any ethics applied (Table 1). We then screened the included medical records to identify ethical themes noted in the records. We performed a final qualitative analysis of the selected prehospital medical records quantifying the qualitative data to identify ethical themes applied in the decision-making process (Table 2).

**Table 1:** The inclusion criteria used by members of the Danish Cardiac Arrest Validation Group during the initial screening

Resuscitation providers documenting the presence of Do-Not-Resuscitate Orders (DNR) or advance directives
Resuscitation providers documenting change in the course of treatment (initiation, termination, continuation, withholding) based on non-clinical reasons (e.g. the appearance of the patient, intangible factors, other factors not of an immediately objectifiable character, etc.)
Resuscitation providers documenting discussion with family or caretakers about an agreed level of care)
Notes where members of the Danish Cardiac Arrest Registry, who collected data, were in doubt about why the resuscitation provider had acted the way they did

**Table 2:** The bioethical principles and identified themes representing the principles

Bioethical principles	Information informing the principles	*n*
Autonomy	Do-not-resuscitate order (patient’s will written on paper)	149
	Patient’s wishes (expressed verbally by a proxy)	165
Non-maleficence/beneficence	Do-not-resuscitate order (Unilateral or unknown origin)	192
	The patient’s prognosis (assessment of length of remaining life or quality of life made by resuscitation providers)	467
	The patients’ prognosis or life quality (assessed by a general practitioner)	57
	Quality of life (assessed by relatives or care personnel)	135
Justice	Future patients (economy or assurance)	0
	Physicians’ considerations regarding others or self	0
	Logistics (Intensive Care Unit or Emergency Medical Services)	0
	Society (economy, assurance, political or cultural values)	0
	Extraneous factors (Relatives’ emotional state, physicians’ heterogeneous interpretation of DNR rules)	15

### Ethical analysis

The guidelines from the European Resuscitation Council contain the basic bioethical principles defined by Beauchamp and Childress [9, 13]:

- Autonomy (Respect the patient’s wishes)
- Non-maleficence (Do not harm the patient)
- Beneficence (Act in the best interest of the patient)
- Justice (Avoid inequalities in treatment)

We broadly defined ethical factors influencing the decision-making process and included any explicit or implicit occurrence of ethical considerations documented in the prehospital medical records. We predefined several categories and subsequently used these in a framework analysis (Additional File 1). We did not discriminate between the two ethical principles, beneficence and non-maleficence, as we consider them ethically related.

### Study context and participants

Denmark has 5.8 million inhabitants [14] and consists of five health regions. The emergency medical services (EMS) are three-tiered and consist of emergency medical technicians (EMT), paramedics, and prehospital physicians in the ground- or helicopter-based emergency care units [15]. The prehospital physician is most commonly an anaesthesiologist who is on-call at the mobile emergency care unit (MECU). The MECU is dispatched as a supplement to the ambulance in all cases of cardiac arrest. In cases where the physician is indisposed or has a long response time to the scene, ambulance crews can reach the prehospital physician via telephone.

In Denmark, only physicians have the authority to declare a person dead. However, paramedics and EMTs may cease or withhold resuscitation attempts after consulting a physician.

Danish legislation does not require specific physician-led decision-making in cases in which patients have a do-not-resuscitate (DNR) order or have verbally refused treatment [16]. Paramedics and EMTs may withhold resuscitation in such cases, and a physician is obliged to withhold or terminate resuscitation if a DNR or a living will is present (Additional File 2). The patient’s physician in charge (most commonly the patients’ general practitioner) may issue a DNR if the patient is critically ill or nearly dying. In this study, these are the orders we refer to as DNRs. Do-not-resuscitate orders issued at the scene of the cardiac arrest by a prehospital physician will not be included in this description. We chose to make this distinction because the knowledge of the patient the general practitioner has and the urgency of the decision the prehospital physician experiences make the two types of do-not-resuscitate orders too different to be considered identical, which is in accordance with Danish law. Professional resuscitation providers (i.e. physicians, EMTs, and paramedics) are required to document the treatment provided in the prehospital medical records [17]. Danish prehospital professionals use a nationwide prehospital electronic medical record system [18]. This system includes a specific form for use in cardiac arrest that forms the basis for the Danish Cardiac Arrest Registry where all OHCAs in Denmark are registered [19]. The cardiac arrest form is a checkbox questionnaire with supplementary free-text fields. None of the text fields are designated specifically to describe the decision-making or the ethical considerations, though one field, entitled notes, can be used to document various information including ethical considerations. In our study, we included entries completed by all prehospital professional resuscitation providers who participated in the cardiac arrest treatment applying the preconception that the physician, having the highest charge at the scene was ultimately responsible for the content of the medical records.

### Data collection and sampling

The Danish Cardiac Arrest Registry is subjected to an annual validation process where a validation group scrutinises each prehospital case that may represent an OHCA. We manually validated 26,732 prehospital electronic medical records with data potentially representing cases of prehospital cardiac arrest from January 1, 2016, to December 31, 2018, and identified 16,495 records with a definitive identification of OHCA. We then re-reviewed these 16,495 validated records. The validation group (consultants, senior registrars, and senior medical students) manually screened the free-text fields in the medical records for documentation of any ethically relevant information by applying broad inclusion criteria. We marked the records with either “ethics” or “DNR” in an electronic template to signal that the inclusion criteria were met. As the screening template was part of the annual validation process, it contained clinical information (bystander CPR, time to CPR initiation, cardiac arrest location, etc.), but also contained a free-text field to register whether the medical record was eligible in this study. To ensure unambiguous validation, we repeated the screening process. The inclusion criteria are presented in Table 1.

### Data analysis

We performed a framework content analysis on the textual elements from the prehospital medical records in the final dataset [20]. The framework approach is useful “where multiple researchers are working on a project, particularly in multi-disciplinary research teams, and for managing large data sets where obtaining a holistic, descriptive overview of the entire data set is desirable”. As such, we deemed this method suitable for our study. The framework analysis consists of five steps: 1. Becoming familiarised with the data by reading and re-reading the whole material, 2. Designing and testing a coding framework to capture relevant themes, 3. Coding of the data 4, Charting in which coded text is collected together in a chart, 5. Through mapping and interpretation, the data is thematically compared and analyzed [20, 21]. After familiarisation, we adapted a preliminary codebook by combining a previously published template [22] with the relevant literature on philosophy and ethics [5]. Two members of the research group, LB and CS (both Masters of Arts and PhDs in philosophy, senior researchers in philosophy, and highly experienced in ethical analysis) independently read the initial 200 medical records and conducted line-by-line open coding to refine the codebook in an iterative process before the remaining records were evaluated. They reached a consensus on final, robust categories through mutual discussion (Additional File 1). We applied the codebook to the medical records but left entries open to new codes identified during the analysis. Through indexation, charting, and mapping, and interpretation, LB, CS, and LM identified key concepts and major themes that were relevant to the basic bioethical principles. We assessed the quality of the medical records. In the assessment of quality, we studied the transparency and the descriptions of the medical records that may be explicit and evident in varying degrees when describing the decision-making process. We considered the medical record of poor quality if the reasons behind the decision-making were not clear and well described.

## Results

The prevalence and characteristics of the available ethically relevant information are reported in two steps: (1) The ethically relevant information related to the bioethical principles (see Table 3 for examples of quotes), and (2) An assessment of the overall quality and transparency of the included medical records.

**Table 3:** Quotes identified in the medical records representing each theme

Theme	Quotes representing the theme
Autonomy	First electrocardiographic analysis shows asystole. It is noted that the patient does not wish for resuscitation to be initiated. For that reason, the treatment is terminated at [time]
	We are informed of a verbal agreement between the patient and relatives of no resuscitation. This is not written on paper and thus resuscitation is continued
	The patient wished to be discharged, [from the hospital] as he wanted to ‘die in his own nest’
Beneficence and non-maleficence	Old weak-looking man, diagnosed with non-specified kidney disease. Placed in an extended care home
	He develops ventricular fibrillation in the ambulance. [We] withhold resuscitation because of advanced age, severe mental illness and the patient’s inability to take care of himself
	The family discourages resuscitation, as the patient has been ill for some time now and was much weakened
	Had Alzheimer's disease, prior history of illness unknown. Do-not-resuscitate wishes from the family and nursing staff
	CPR is immediately initiated, at the paramedic’s arrival asystole despite CPR, it turns out that “no resuscitation” was determined in advance, death is declared at [time]
	We find a folder in [the patient’s] house, this states loud and clear that the patient was in the terminal phase of a cancer disease!!! Therefore, the patient should not have been resuscitated, poor communication from the nursing staff
Justice and extraneous factors	There is a letter on no attempt at CPR. Not signed by a physician and the original text has been altered. [It] is not valid in the situation
	At our arrival, the nurse is performing CPR. [The] family does not want the patient resuscitated. This wish cannot be granted, as there is not a written statement from the general practitioner. We initiate CPR. At the same time, [the family] calls the general practitioner, who asks me to terminate resuscitation, to which I have to say no to, as there, once again, does not exist a written statement regarding this
	We retreat from the scene when the son gets infuriated. Police are on their way. Resuscitation has not been initiated at any time
Best practice examples	The patient has been in palliative care, has in-home nursing staff, who has a document that shows the patient does not wish for resuscitation to be initiated. The husband clearly expresses that the patient did not want resuscitation in case of cardiac or respiratory arrest. The physician at the emergency dispatch centre is contacted and agrees to withhold resuscitation based on the medical history and the patients’ wishes
	Resuscitation attempt and the potential subsequent treatment will be with no chance of survival, and in agreement with the patient’s wishes, resuscitation will not be attempted

### Ethically relevant information and bioethical principles

**Fig. 1:**
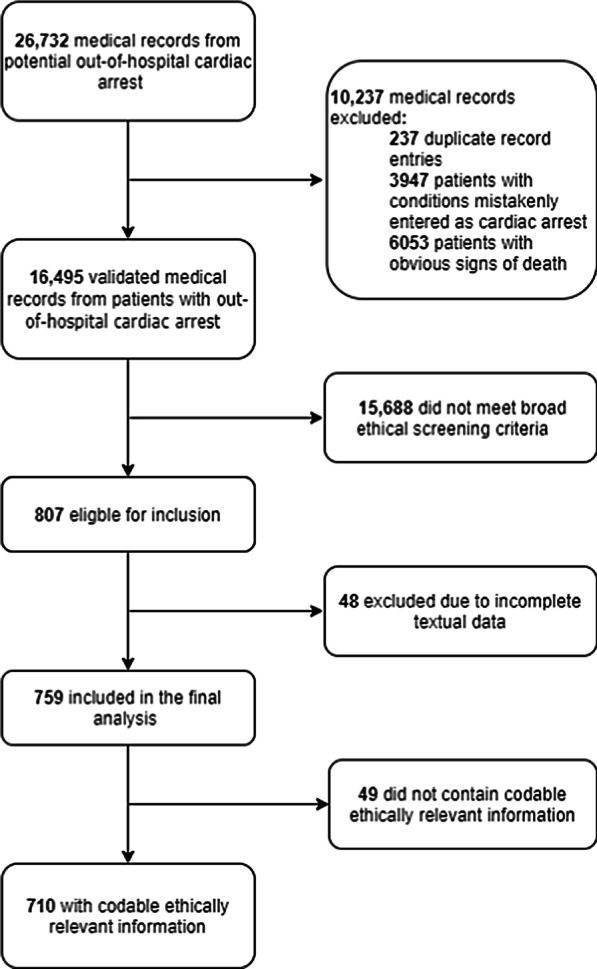
Flowchart of the inclusion process

Within the 16,495 medical records concerning OHCA, we identified 759 (4.6%) medical records that, according to the validation group, might contain ethically relevant information (Table 1). Following review by the experts in ethics, 710 (4.3%) medical records were found to have one or more entries of ethically relevant information while 49 medical records were not considered to contain ethically relevant information (Fig. 1). We identified one new code “The patients’ prognosis or life quality (Assessed by a general practitioner)” during the analysis process that was not included in our codebook.

We identified 1180 statements with ethical relevance in the 710 selected medical records. The content based on the different categories of basic bioethical principles is outlined below (see Table 2). See Table 3 for examples of quotes.

### Autonomy

Information influencing the decisions based on patient autonomy included both DNRs (n = 149) and patient wishes and views on resuscitation (n = 165) (Table 2). We defined “patient’s wishes” as available statements or previous actions from the patients indicating their wishes concerning termination of or refraining from resuscitation. Most commonly, relatives, nursing staff, or the general practitioner verbally communicated the patients’ wishes to the resuscitation providers (Table 3). Some patients had explicitly declared their wishes through either a living will or a DNR. In other cases, the patients had implicitly expressed their wish not to prolong their lives by refusing to eat or to receive further medical treatment before the cardiac arrest. Resuscitation providers refrained from resuscitation attempts in most cases where the patient’s wishes were documented. Some resuscitation providers accepted a verbal statement from relatives or nursing staff as a reason for terminating resuscitation; others continued resuscitation despite explicit dissent from relatives. In some situations, the resuscitation providers questioned the legal validity of the verbal statements and required a prehospital physician to decide whether was to be honoured.

### Beneficence and non-maleficence

We considered that the bioethical principles of beneficence and non-maleficence were conveyed by the themes “life expectancy” and “quality of life,” as assessed by resuscitation providers; the “quality of life” as assessed by relatives or care personnel; and by DNRs. “Life expectancy” influenced resuscitation in 467 of the 710 medical records (65.7%; 95% CI 62.2%–69.3%) (Table 2). Most commonly, the resuscitation providers implied that the patient would face a poor prognosis should the resuscitation be successful (Table 3). The statements included a global approach to the patient’s condition, including clinical features, comorbidities, and the level of daily functions. In many cases, resuscitation was deemed “futile” and terminated. In addition to the descriptions of the quality of life made by the physicians, in 135 cases, other resuscitation providers registered their perceptions of the patient´s quality of life. In these statements, the resuscitation providers described the relatives’ wishes and thoughts on the patients’ potential benefit from the resuscitation attempt. At times, these opinions formed part of the decision-making process. In other cases, the resuscitation providers continued CPR disregarding the relatives’ explicitly stated opinions that the resuscitation attempt was not in the best interest of the patient. When present, nursing staff could state their opinion regarding resuscitation, explicitly or implicitly through actions such as refraining from resuscitation attempts before the arrival of the emergency service providers. In 57 cases, the patient’s general practitioner influenced the decisions either by being physically present or by telephone.

Almost half of the 710 medical records included in the final analysis mentioned a living will or a DNR issued by the patients’ physician in charge (e.g. their general practitioner). In 192 medical records, the DNRs were considered relevant to non-maleficence/beneficence as the order was unilateral, that is, written by a physician with or without the patient’s consent. The resuscitation providers mentioned the presence and, in some cases, the absence of a DNR if they deemed the resuscitation attempt inappropriate. In some cases, where resuscitation was initiated despite a DNR, the DNR was not presented to the resuscitation providers because the nursing staff or relatives were not present at the scene or were unaware of the DNR. This frustrated the resuscitation providers.

### Justice and extraneous factors influencing equality in care

We defined content relevant to the basic principle of justice in considerations about the consequences for future patients, consequences of the physicians’ working conditions (e.g., sleep deprivation, safety concerns, fear of legal implications, etc.), logistics, or concerns about overall societal welfare. However, we did not find relevant content in any of these categories. Nonetheless, we detected content relevant to justice in extraneous factors influencing the decision-making, such as the influence of the relatives’ emotional state on decision-making in 15 medical records (Table 2). We identified possible justice bias in the heterogeneous interpretation of DNRs and the variable influence of the presence of relatives, where resuscitation providers questioned the validity of the statements obtained from the relatives in some cases and, as a result, continued resuscitation despite a DNR (Table 3). In other cases, a verbal statement from relatives was considered a sufficient reason to terminate resuscitation. The medical records contained cases where interference by relatives did overthrow the resuscitation providers’ initial decision regarding continuation and discontinuation of resuscitation. In these cases, verbal or physical abuse was one of the reasons for changing decisions, and resuscitation providers were occasionally forced to leave the scene despite initially deciding to resuscitate a patient.

### The overall quality of ethical documentation

In general, the descriptions of ethical reasoning in decision-making were vague and unclear. In most cases, it was only possible to infer indirectly that ethical principles had been considered. Descriptions of DNRs and their roles in the decision-making were particularly vague or unclear. It was often unclear who had issued the DNR, and whether the patient had been informed and had agreed with the decision. A few cases, though, explicitly described the full range of relevant factors in the decision-making (Table 3). In these “best practice” examples, the resuscitation providers stated relevant considerations in a short, adequate, and coherent argument with a clear conclusion.

## Discussion

This Danish study shows that about one in twenty OHCA medical records contains ethically relevant considerations and that documentation on the decision-making in prehospital resuscitation is vague and generally unclear. However, four themes that reflect the bioethical principles of autonomy and beneficence/non-maleficence were identified: Patient wishes and perspectives on life; Relatives’ wishes and perspectives on the patients’ life; Healthcare professionals’ opinions and perspectives on resuscitation; and DNR orders.

The paucity of ethically relevant information in the medical records confirms the findings of another study [22], where only 62 (4.9%) of the 1275 OHCA patients had ethical considerations documented in their medical records. There are at least three possible explanations of the missing documentation: Based on mere clinical factors influencing the chance of success in resuscitation, the absence of ethical considerations may be appropriate, as the predominant indication for lack of treatment may have been purely clinical. The assessment by the healthcare professional may have been that successful resuscitation was impossible [10, 22]. Prehospital resuscitation providers work in stressful environments under considerable time constraints leaving little time for in-depth documentation [23, 24] However, our finding of “best practice” examples indicate that accurate and transparent documentation is feasible in the prehospital environment.

The lack of documented ethically relevant information may be a result of a simplified evidence-based approach to medical treatment and documentation. Evidence-based medicine aims to achieve objectivity in medical treatment with the help of guidelines, leaving little room for non-quantifiable considerations [25]. This notion is supported by in-hospital research from an intensive care unit, where Ratnaplan et al. reported that intensivists usually document the patients’ treatment plan but rarely include its likelihood of benefit or include considerations regarding the patients’ previous wishes [26]. Furthermore, some physicians may believe that “subjective” considerations do not have an appropriate place in objective medical records. In Denmark, the documentation of out-of-hospital resuscitation adheres to the Utstein guidelines. These guidelines do not mention ethically relevant information as part of standard reporting on prehospital resuscitation [27]. Likewise, the Danish prehospital electronic medical record system does not include checkboxes or free-text fields concerning non-clinical or ethical considerations. However, this lack of ethically relevant medical reporting and documentation does not necessarily reflect the actual, at-the-scene decision-making practice [28]. Several qualitative studies support this theory and find that ethics such as ethical dilemmas *are* a part of decision-making in prehospital resuscitation [8, 29, 30].

Nevertheless, the lack of documentation of ethical aspects in decision-making may pose a problem. Post-event peer scrutiny is impossible if documentation on various aspects of decision-making is opaque or non-existent. Healthcare professionals’ personal beliefs and values may influence decision-making in resuscitation [31–34]. In a qualitative study, Brandling et al. reported that personal factors such as pre-existing influences, whether factual or perceptual, primed the EMS providers' decision-making [34]. Thus, there is a risk of heterogeneity in prehospital resuscitation decisions in a work environment where physicians are the sole decision-makers without the possibility for deliberation with peers. This may lead to overtreatment at one end of the spectrum and therapeutic nihilism or unconscious bias concerning for example race, age, or personal capabilities at the other [35]. As previously stated, prehospital physicians have the possibility of consulting the ambulance crew, but in a non-randomised trial, only 48% did so [5]. The prehospital physician has the ultimate responsibility for the treatment. However, good teamwork skills may prevent feelings of uncertainty, which in turn may facilitate good clinical decision-making and increase patient safety [36]. When team discussions do take place, resuscitation providers consider consensus within the crew to be an important support in decision-making [34]. On the other hand, ethical dilemmas may arise if opinions within the crew diverge [37]. Mandatory documentation of explicit ethical considerations could enhance the quality of the decision-making process and ensure accountability by enabling post-event scrutiny.

We found that ethically relevant information primarily was related to the basic bioethical principles of autonomy and non-maleficence/beneficence. This finding is in line with previous research [4, 6, 11, 38, 39]. The assessment of the patients’ quality of life and the remaining length of life were predominant factors identified in this study. We found that the documentation of DNR orders was vague and non-transparent. In half of the DNR orders, it was impossible to determine whether the DNR was an expression of the patient’s autonomy, a decision made by a physician alone, or a joint decision by the two stakeholders. Resuscitation providers may consider the act of respecting a DNR order as an act of respecting patient autonomy, but the patient’s general practitioner can issue a DNR based on the patient’s general health status. Danish law states that physicians should discuss these decisions with the patient, although the physician has the final say when issuing a DNR. Thus, a DNR ultimately does not require patient consent [16]. The resuscitation providers’ insecurity in dealing with advance directives and end-of-life decisions may result in non-transparent descriptions and heterogeneity in the interpretation of DNR orders [40, 41]. Concerns of legal responsibility may be reflected in the documentation and descriptions of DNR orders [42]. The lack of clarity concerning the role and content of DNR orders is problematic given the authoritative status they have in OHCA. DNR only represents one aspect of the patients’ wishes: The wish for no resuscitation. The other aspect is patients who wish to be resuscitated. Laakkonen et al. show half of their patients, with a mean age of 80 years, would prefer CPR [43]. Thus, some elderly patients that wish to be resuscitated will have their treatment terminated on-site thereby not honouring their wishes. These cases were not represented in our study.

Resuscitation providers documented the relatives’ emotional state that, in some cases, influenced decision-making. The presence of relatives does not influence prehospital resuscitation in general [44], but it may influence treatment when their opinions on resuscitation conflict with the resuscitation providers' legal obligations. Previous studies describe the complex and challenging situation the relatives face when their loved one suffers from cardiac arrest [45, 46]. De Stefano et al., investigating the presence of family members during resuscitation, found that relatives may experience resuscitation as an aggressive overtreatment [45]. This perception can explain the relatives’ frustration described in our study when resuscitation providers, against family wishes, treat the patient as obligated by law. However, we found few examples of this situation. It may lead to the continuation of futile CPR when resuscitation providers balance between fear of legal repercussions and/or the relatives’ perceived expectations, demands, and reactions on one hand and empathy for the patient’s wishes at the end of their life on the other [34]. Bremer et al. [47] found that some EMS providers continue obvious futile CPR because they feel inadequate to meet the relatives’ expectations. In a study by Helicser et al., it was found that resuscitation providers face ethical dilemmas when the wishes of family members and those of the patient are incompatible [48]. The family's wishes may be contradictory to the patient’s wishes. Even though family wishes and surrogate decision-making can be useful in certain situations, previous research has identified weaknesses in this concept [49]. The patient’s preferences regarding resuscitation may change over time but are less likely to do so if they are filled out in DNRs [49]. Resuscitation providers should honour patient autonomy, and avoid a continuation of CPR if it is against patient wishes [50].

Thus, we cannot conclude that prehospital decisions are devoid of ethical considerations despite the absence of documentation in the medical records. However, further research utilising qualitative methods may elucidate ethical considerations in resuscitation further and increase transparency regarding these life and death decisions that are usually made at the prehospital scene without deliberation with peers.

### Limitations

One of the limitations of this study is that the search strategy did not allow for a distinction between statements made by EMTs, paramedics, or physicians. However, the prehospital physician is ultimately responsible for the decision-making processes and thus ultimately responsible for the entries in the medical records. Thus, one can assume that the documented ethical considerations are part of the decision-making process, regardless of who documents them. Another limitation is that the members of the validation group who performed the initial screening for ethical content may have overlooked medical records with statements that experienced philosophers would have classified as ethical considerations.

## Conclusions

Only a small number of prehospital medical records from cases of out-of-hospital cardiac arrest contained ethically relevant information. This information tended to be vague and unclear. “Best practice” examples, however, indicate that documentation of ethically relevant information in decision-making is feasible. Previously, it has been shown that medical decisions concerning termination of treatment in cardiac arrest may vary between treatment centres [32, 51]. Furthermore, it has been shown that the factors determining resuscitation are often a complex combination of validated criteria and factors resulting from an intuitive perception of the outcome [4]. As this leaves room for unequal treatment, more transparent and systematic documentation in medical records is necessary, given that decision-making on resuscitation in most cases has an irreversible outcome. We thus propose a reworking of the medical record systems leaving room for specific ethical considerations.

## Supplementary Information

**Additional file 1.** Final codebook displaying factors for decision making other than purely medical and legal considerations, including what is traditionally seen as ethical values as well as political and emotional considerations.

**Additional file 2.** Excerpt from the Danish legislation regarding resuscitation and omission of resuscitation.

## Data Availability

The datasets generated and analysed during the current study are not publicly available due to them containing information that could compromise patient privacy but are available from the corresponding author on reasonable request.

## Abbreviations

OHCA: Out-of-hospital cardiac arrest
EMS: Emergency medical services
EMT: Emergency medical technician
DNR: Do-not-resuscitate order
CPR: Cardiopulmonary resuscitation
