# Supplementary material for: Documentation of ethically relevant information in out-of-hospital resuscitation is rare: a Danish nationwide observational study of 16,495 out-of-hospital cardiac arrests
Source: BMC Med Ethics. 2021 Jun 30;22:82. doi: 10.1186/s12910-021-00654-y (PMC8247191; doi:10.1186/s12910-021-00654-y)
Supplement: Supplementary file 1 — Additional file 1. Final codebook displaying factors for decision making other than purely medical and legal considerations, including what is traditionally seen as ethical values as well as political and emotional considerations. [file 12910_2021_654_MOESM1_ESM.docx]

Additional file 1: Final codebook displaying factors for decision making other than purely medical and legal considerations, including what is traditionally seen as ethical values as well as political and emotional considerations.

| **Overall categories** |  | **Sub-categories** |
| --- | --- | --- |
| **Patient** |  | a. Do-not-resuscitate order |
|  |  | b. Wishes and outlook |
|  |  | c. Life expectancy (length or quality) |
| **Relatives** |  | a. Emotional state |
|  |  | b. Wishes and outlook |
| **Bystanders** | Nursing staff | a. Emotional state |
|  |  | b. Outlook |
|  | General practitioner | b. Outlook |
|  | Emergency medical dispatch centre | b. Outlook |
| **Future patients** |  | a. Economy |
|  |  | b. Assurance |
| **Physician** |  | a. Considerations regarding others |
|  |  | b. Considerations regarding self |
| **EMT** |  | a. Logistics |
|  |  | b. Emotional state |
| **Intensive Care Unit** |  | a. Logistics and economy |
| **Society** |  | a. Economy |
|  |  | b. Assurance |
|  |  | c. Political/Cultural values |
