# Supplementary material for: Documentation of ethically relevant information in out-of-hospital resuscitation is rare: a Danish nationwide observational study of 16,495 out-of-hospital cardiac arrests
Source: BMC Med Ethics. 2021 Jun 30;22:82. doi: 10.1186/s12910-021-00654-y (PMC8247191; doi:10.1186/s12910-021-00654-y)
Supplement: Supplementary file 2 — Additional file 2. Excerpt from the Danish legislation regarding resuscitation and omission of resuscitation. [file 12910_2021_654_MOESM2_ESM.docx]

**Excerpts: Executive order no. 9934. Date issued: 29th October 2019**

**Danish Patient Safety Authority Order no. 3­3010­272/1**

**Executive order regarding resuscitation and omission/opt-out of resuscitation.**

1. Introduction

The purpose of this executive order is to ensure that patients are granted a dignified end-of-life by supporting opting out of resuscitation attempts if this is medically relevant. The executive order is relevant for all patients and applies no matter where the treatment takes place. As such, it is valid in all instances occurring in hospitals, nursing homes, private institutions, private homes, etc.

2. Resuscitation should be attempted as a general rule in any patient with presumed cardiac arrest. There are five exceptions. If the healthcare provider is in doubt whether an exception applies, resuscitation should be commenced.

Exception no. 1: Special situations where other than doctors can ascertain that the patient is dead

1. Only a physician has the authority/competence to declare a patient dead. Others may, however, declare a patient dead if there are obvious signs of death: Extensive putrefaction; obvious mortal injuries (i.e. a crushed skull).
2. In case of a witnessed and predicted death in a patient provided a nurse or another caregiver who has been involved in the treatment is present.
3. Death occurs while the patient is alone in his home and he has been a patient in the home care as part of terminal care. In those cases, a nurse, a social and health worker or other caregivers who have been involved in the care and treatment of the patient, may declare that the patient has died.
   Situations on litra b) and c) prescribes that a physician is involved in the treatment.

Exception no.2: A physician opts out of resuscitation attempts after encountering a lifeless patient.

1. Except in cases of hypothermia, a physician may declare that a patient without obvious or reliable signs of death is dead and resuscitation attempts are futile. This declaration should be based on the physician’s knowledge of the patient or the information provided. This information may be handed over by telephone and the physician is not necessarily required to be physically present.

Exception no. 3: The physician in charge of treatment has decided in advance to refrain from resuscitation

1. The physician in charge of the treatment may decide not to attempt resuscitation stop if the patient is seriously ill or dying, and further treatment is considered futile. The physician must

involve the patient in these deliberations, but the physician has the authority to make this decision. The patient cannot demand to be resuscitated.

1. The patient is unavoidably dying.
2. The patient is seriously incapacitated to such an extent that he/she is unable to take care of himself physically and mentally and is cut off from any meaningful human contact
3. The patient is in a condition where attempts at resuscitation may lead to survival, but where the physical consequences of the disease or the treatment are judged to be very serious and to cause the patient much suffering.

Situations on litra a) through d) requires all other healthcare professionals to comply with the decision made by the physician responsible for treatment to refrain from resuscitation attempts. The decision to opt-out is valid until the physician responsible for the treatment decides otherwise. If the patient's condition improves significantly, the physician in charge of treatment must be contacted to assess whether there is a need to change the previous decision.

If the patient suffers from a prehospital cardiac arrest and prehospital personnel has been called, the healthcare professionals present must inform the prehospital personnel of this decision, as far as possible by presenting the physician´s written notification of the opt-out.

Exception no. 4: During the present/current disease, the patient has decided that the caregiver should opt-out of resuscitation attempts.

1. The doctor in charge of treatment must decide whether the patient is competent and 18 years of age or older and ensure that the patient is adequately informed to make the decision.
2. Patients of the age 15 – 17 years may opt-out of resuscitation attempts if the attending physician has ensured that the patient has understood the consequences of the decision. Furthermore, the physician is obliged to give the custodians the same information as the patient has received.
3. The patient's decision to opt-out of resuscitation attempts is only valid in connection with the current disease situation.
4. If the patient's condition improves significantly, the situation should be regarded as a new situation, meaning that the decision to opt-out of resuscitation attempts is no longer valid.

Exception no. 5: The patient has created an advance directive or living will stating that resuscitation should not be attempted.

1. A patient may have indicated in a living will that he or she does not want life-prolonging treatment, including resuscitation attempts, in certain specified situations. The will becomes valid and binding if a physician assesses the patient as being permanently incapacitated. The patient´s incapacity is thus a precondition for the will to become valid.
2. The living will declare that the patient’s wishes for the caregiver to refrain from resuscitation applies only to the current admission to hospital or the current course of treatment. In general, weakening because of old age alone therefore does not allow for opting out of resuscitation attempts.
